# The consequences of the economic crisis in radiology

**DOI:** 10.1007/s13244-015-0434-9

**Published:** 2015-10-01

**Authors:** European Society of Radiology (ESR)

**Affiliations:** European Society of Radiology (ESR), Neutorgasse 9/2, 1010 Vienna, Austria

**Keywords:** Economic crisis, Radiology, Teleradiology, Radiology market, Radiology reimbursement

## Abstract

**Abstract:**

The effects of the economic crisis have led to complex problems in radiology. The crisis has led to a reduction in the turnover of imaging equipment. This reflects on the quantity and quality of output, an aspect which is worsened by the contraction of the radiology market, late payments on supplies, and competitive procurement of medical goods centralized on a regional or national level. Many local and national institutions have operated with significant reductions of reimbursement for procedures, forcing a reorganization of facilities, manpower, and equipment. The reduction in operating margins of the industry has resulted in a reduction of invested capital for projects of industrial R&D and direct or indirect sponsorship. The quality of care will be affected with less comfortable conditions, reduction of local availability of radiologists, and failure to invest in lower dose equipment to control population medical radiation exposure. The crisis resulted in a reduction in the number of graduates in medicine and scholarships for specialization induced by linear cuts will result in a drastic reduction of radiological specialists. This will favour the development of teleradiology services, with the risk of accelerating the demedicalisation of radiology departments, and isolation of the professionals.

***Main messages*:**

• *The economic crisis has led to reduction in the turnover of imaging equipment.*

• *The economic crisis has led to reductions of reimbursement for procedures.*

• *The economic crisis has led to reductions in operating margins of the industry.*

• *The economic crisis has led to contraction of quantity and quality of output.*

• *The economic crisis resulted in demedicalisation of radiology departments and isolation of professionals.*

## Background

The demand for healthcare is increasing across Europe, driven by a number of factors. This is partly due to developments in medical sciences and technology, but the biggest driver is probably the marked demographic changes that are occurring in the early twenty-first century. The aging population is the main concern of many economical discussions, not just healthcare. Not only are there increased numbers of people over retirement age, but these individuals are living longer as well. It is this population that carries the highest burden of the most common healthcare diseases, such as cancer and cardiovascular disease. By the end of 2020, in many western countries, the working-to-retired person ratio will be 3 to 1. This will put huge pressure on other social security budgets besides healthcare expenditure. Almost all European countries have, therefore, developed healthcare reforms in an effort to constrain the constant increase of healthcare expenditures in the last 10 years [1, 2].

This challenge has been made even greater by the recent worldwide economic crisis. Some governments have responded with policies aimed at reducing public spending on healthcare. Countries implementing healthcare reforms have endeavoured to moderate the growing budgets for healthcare services, rationalized the benefit packages while focusing on the medical service rendered, increased the share of health expenditure paid by private households, and implemented wide-reaching reforms in the pharmaceutical market.

Concerns about the potential impact of the financial crisis on the ability of countries to achieve health system goals led the World Health Organization’s (WHO) Regional Committee to adopt the 2009 resolution “Health in times of global economic crisis: implications for the WHO European Region (EUR/RC59/ R3a)”. The resolution urged member states to ensure that their health systems will continue to protect the most vulnerable, to demonstrate effectiveness in delivering personal and public healthcare services, and to behave as wise economic actors in terms of investment, expenditure, and employment [3–7].

The possible health policy responses to the financial crisis and other health system challenges include three key dimensions: health expenditure options, policy tools, and outcomes.

- Policy makers may face pressure to maintain, decrease, or increase current levels of public expenditure on health. With any of these options they could also reallocate funds within the health system in order to enhance efficiency, with a possible shift from the focus on diagnosis and treatment to investment in disease prevention.
- A range of policy tools can be used to alter expenditure levels.
  The level of contributions for publicly financed care (the size of the national health budget, social insurance contributions and transfers from the health budget, fiscal policy, and private expenditure on health in the form of user charges and/or private health insurance).
  The volume and quality of publicly financed care (the statutory benefits package, population coverage, and non-price rationing such as waiting times).
  The cost of publicly financed care (the price of medical goods, salaries and motivation of health sector workers, payments to providers, overhead costs, and reconfiguration or coordination of care).
  The control of the size of healthcare economics in a country (strategic planning or provision and demand control; e.g., provision control will be achieved by having a control on healthcare investments, demand control by waiting lists, and co-payment issues).
    In many cases, policies will affect more than one of these factors.
- When making decisions, policy makers need to consider the impact of any proposed reforms on health system goals, including improving health, financial protection, efficiency, effectiveness, equity, quality, responsiveness, transparency, and accountability. Unfortunately, in situations such as economical crisis, financial protection and efficiency become the most important goals, sometimes other items are overlooked [8–11].

## The economic crisis in radiology

Radiology is considered by many to be at the heart of healthcare. Advances in sophisticated technologies have extended its application scope to every organ, offering not only essential services in diagnosis, but now also in therapy. Radiology is constantly evolving both technologically and also in its clinical application. The number of performed procedures is growing, even if the market for new equipment is currently slowing down due to the economic crisis.

Europe includes a number of countries with diverse economies, existing healthcare systems and infrastructures, needs, and spending, making it a “multispeed” entity. On the one hand, countries mainly in the south of Europe are slowing down their radiology investments due to the economic crisis, whilst in other parts of Europe a rather sustainable growth is observed. The assessment of the effects of the economic crisis in European radiology is, therefore, very complex in terms of structural, organizational, clinical, professional, cultural aspects of this discipline. The relationships with traditional stakeholders have been modified, redesigned, and adapted to the new needs and growth prospects of macro- and microeconomics.

### Structural effects

The crisis has led to a reduction in the turnover of imaging equipment resulting in a higher than usual level of aging of technological equipment, at a time when technological developments are still increasing. The plan for the purchase of new technologies will slow down with a level of obsolescence constantly increasing.

This slowdown in equipment replacement is not only the direct result of shrinking budgets, but also the consequence of adaptive strategies leading to a better use of resources. Radiology based on highly technical hardware and diagnostic pathways offers a fertile ground to workflow standardization resulting in productivity gains. Efficiency plans focus on a variety of measures including: merging of structures, sharing of equipment, closing down excess capacity equipment, policies of equipment upgrade, patient throughput optimization, and extension of opening hours. This has led in some circumstances to a reduction of the number of necessary new units. There is also a drive to rationalize healthcare access, which in radiology is focused on reducing unnecessary imaging that does not have a clear impact on patient care.

At the same time, routine maintenance programmes of equipment may be delayed, running the risk of physical deterioration of equipment. This can result in equipment failure linked to a lack of plans for substitution or replacement.

The reduction of resources for maintenance of the facilities, diagnostic rooms, air conditioning systems, waiting rooms for the patients, represents an epiphenomenon of the more general problem, but is able to influence negatively the routine activities of the department.

The general atmosphere of healthcare reforms, increased control of healthcare expenditures, downward revision of tariffs, increased controls by the paying agencies creates uncertainty about future revenues, and reluctance to medium-term investment.

This has negative effects on the quantity and quality of output in terms of radiological procedures provided, by adversely affecting the financial and human resources of the department, self-fuelling the crisis already in place.

Impacts on quality are because, especially in radiology, new equipment offers higher imaging quality and reduced radiation exposure, owing to the improvement of technologies using X-rays or to the substitution of non-ionizing technologies (e.g. MR imaging).

### Budgeting

Many local and national institutions have operated with significant reductions of reimbursement (>25 %) for procedures which has forced a reorganization of facilities, manpower, and equipment. Moreover, in some instances, the parallel reduction of demand means that the mechanism for the reorganization of resources becomes even more critical.

This reduction in budget for radiology departments can lead to a biphasic effect on volumes within a fee-for-service model. There can be an initial tendency to increase the volumes to compensate the diminution in revenues resulting from the reduced procedural reimbursements, followed by a trend to reduce the volume of procedures requested, due to the attention paid to appropriateness. However, the parallel increase in the number of patients due to the inexorable rise of ageing, chronic diseases, and defensive imaging provides a see-saw effect; as a result, global volumes are not expected to drop.

In systems where overall reimbursement is guaranteed by the government, there is a tendency to increased used of low cost techniques, such as ultrasound and plain radiography, to attempt to substitute for the higher costs of higher tech procedures such as MRI and CT. This is one of the explanations for the disproportionate place of ultrasound and radiographs in some countries, in comparison with more sophisticated imaging techniques.

### The crisis and the industry

The economic crisis that has contracted the radiology market (equipment, devices, equipment supplies, drugs, etc.), late payments on supplies, and competitive procurement of medical goods centralized on a regional or national level led to significant reduction of prices and volumes in the market. As a consequence of this, the reduction in operating margins of the industry has resulted in a reduction of invested capital for projects of industrial R&D and direct or indirect sponsorship for projects, clinical trials, and updating courses for the operators. Also, the level of service quality has been affected due to cost reduction programmes in the industry. This directly impacts radiology by delayed response time, longer waiting times for spare parts, etc.

An additional impact on radiology results from the consolidation of the players, as well as comprehensive packages provision (one single vendor furnishing all the equipment of a facility), which may exacerbate the dependency of the profession on the industry. In addition, in the present context of waste reduction, niche products tend to be discontinued.

### The crisis and the patients

The quality of care that patients and the public receive in radiology services will be affected in a number of ways by these changes. Less comfortable conditions for the patients may be offered due to both ageing of the facilities and equipment and geographical distancing of healthcare supplies, as the consequence of merging of structures to offer economies of scale. The effects on the radiologist workforce, in particular centralization of workforce and outsourcing, will result in reduction of local availability of the radiologist to the patient and their referring clinician. Delayed replacement of older systems may not only result in reduced workflow efficiency, but also may result in poor optimization of radiation dose to the patients and, therefore, potential increased medical radiation exposure for the community. A reduction of the operational equipment base compared to the local needs carries the risk of longer waiting lists and more frequent postponement and rescheduling of procedures. The question of which patients have to be prioritized could become a daily reality having potential negative impact on screening, diagnostic, and therapeutic imaging. A survey sent by the European Society of Radiology (ESR) at the end of 2013 to each of its affiliated European national radiological societies^1^ showed that 58 % out of the 33 responders related a restriction in access to central funding or availability difficulties.

### The crisis and the “profession of radiology”

The potential reduction in the number of graduates in medicine and scholarships for specialisation induced by linear cuts that many governments are implementing will result in a drastic reduction of radiological specialists in the next 5 years. This perception was also supported by the ESR survey in which 58 % out of the 31 responders considered not to have enough radiologists for their current necessities and 55 % out of the 30 responders replied that they would not have enough radiologists in training to serve their respective nations.

This phenomenon threatens clinical radiology as a specialty, since other professionals (orthopaedists, cardiologists, and vascular surgeons) perform diagnostic and interventional procedures where radiologists are lacking. This runs the risk of a reduction in quality control due to the lack of formal training programmes for these professionals as well as the risk of inflation of the procedure volumes due to self-prescription.

In parallel, in some countries, the continuing increase in the number of radiographers with rates more than double that of radiologists will exhibit the risk of progressive demedicalisation of radiological diagnostic procedures with the delegation to non-physician operators (sonographers, radiographers) for performing ultrasound or X-ray due to lower costs.

Another cause for concern is the perspective of income reduction which may reduce the attractiveness of the specialty for new students, compared to other specialties.

The use of business management systems in medical practice and the continuing drive for efficiency has resulted in an important individual workload increase, which is reported in many countries. This focus on reporting workload threatens other important roles of the radiologist, resulting in radiologists having less time for contact with patients and their clinical colleagues. The importance of direct communication with referring clinicians cannot be overemphasised as such discussions aid in improving the patient pathway by correct use and interpretation of imaging tests and, therefore, improved outcomes. The loss of such important aspects of the radiologist’s role has often been replaced by other more administrative tasks, to face competitors and organizational changes, and the loss of some of their personal independence due to the increase in regulation and individual productivity monitoring. All these changes can result in a loss of job satisfaction for the radiologist. These changes run the risk of demotivation and burn-out of the radiology workforce as the increased use of technology moves radiology away from a professional resource to a diagnostic commodity.

The adaptive strategies to face a shortage of radiologists will favour the development of teleradiology services, with the risk of accelerating this demedicalisation of radiology departments and isolation of the professionals.

Thirty-one respondents to the abovementioned survey indicated that the main challenges for radiology in their country were workload increase (77 %), equipment ageing (55 %), shortage of staff (55 %), turf battles (48 %), drop-down in reimbursement (45 %), price competition due to teleradiology (26 %), delegation of tasks from radiologists to technicians (26 %), and reduction in the number of staff positions (16 %).

## Conclusions

The current economic crisis has come at a time of major change and increase in demand for radiological services. This has also coincided with a major change in the way radiology is practiced, predominantly driven by the IT revolution of Picture Archive and Communication Systems (PACS).

The combination of these factors has led to a mismatch between the demand for radiology and the resources available to deliver high quality, safe services.

To avoid the situation deteriorating further, the following actions need to be taken:

- Appropriate use of existing services informed by evidence-based guidelines. This should not only improve the patient pathway through radiology services, but will lead to other efficiency improvement elsewhere in healthcare. By moving the diagnostic process earlier in the patient pathway, there are potential benefits of patients being reassured and avoiding unnecessary further referrals and treatments. In addition, those with disease will have the opportunity of earlier diagnose and earlier treatment. Such guidance will also ensure that patients are exposed to the lowest dose of radiation required to diagnose and monitor their disease.
- Robust equipment replacement programmes that take into consideration optimization of radiation dose and improved efficiencies
- Coordinated workforce plans that include information based predictions of future radiology workforce in order to maintain qualitative and quantitative standards of radiological procedure.

## Footnotes

1 Responses came from Albania, Austria, Belgium, Bosnia and Herzegovina, Bulgaria, Croatia, Cyprus, Denmark, Estonia, Finland, France, Germany, Great Britain, Greece, Hungary, Iceland, Ireland, Israel, Italy, Latvia, Luxembourg, Malta, Norway, Poland, Portugal, Russia, Serbia, Slovenia, Spain, Sweden, Switzerland, Turkey, and Ukraine.
